# The New England Farmer

**Published:** 1862-08

**Authors:** 


THE NEW ENGLAND FARMER.—This is an agricultural journal published at Boston, edited by Simon Brown and H. F. French. It is one of the most valuable agricultural journals in this country. It should be in the hands of every farmer. It gives descriptions of all varieties of soils, their adaptability for certain products, and the best method of culture and treatment; it also gives descriptions and representations of all new and improved farming implements. It also gives a large amount of experience and direction in regard to raising and the treatment of stock; all of which is fraught with great interest to every farmer.

It is published monthly, at $1.00 per year. It is certainly the best investment of that amount any one could make. T.

